# The Gut-Brain Axis in Multiple Sclerosis. Is Its Dysfunction a Pathological Trigger or a Consequence of the Disease?

**DOI:** 10.3389/fimmu.2021.718220

**Published:** 2021-09-21

**Authors:** Benedetta Parodi, Nicole Kerlero de Rosbo

**Affiliations:** ^1^Department of Neurosciences, Rehabilitation, Ophthalmology and Maternal-Fetal Medicine (DINOGMI), University of Genoa, Genoa, Italy; ^2^TomaLab, Institute of Nanotechnology, Consiglio Nazionale delle Ricerche (CNR), Rome, Italy

**Keywords:** dysbiosis, intestinal permeability, neuroinflammation, experimental autoimmune encephalomyelitis, enteric nervous system, vagus nerve, probiotics, short-chain fatty acids (SCFA)

## Abstract

A large and expending body of evidence indicates that the gut-brain axis likely plays a crucial role in neurological diseases, including multiple sclerosis (MS). As a whole, the gut-brain axis can be considered as a bi-directional multi-crosstalk pathway that governs the interaction between the gut microbiota and the organism. Perturbation in the commensal microbial population, referred to as dysbiosis, is frequently associated with an increased intestinal permeability, or “leaky gut”, which allows the entrance of exogeneous molecules, in particular bacterial products and metabolites, that can disrupt tissue homeostasis and induce inflammation, promoting both local and systemic immune responses. An altered gut microbiota could therefore have significant repercussions not only on immune responses in the gut but also in distal effector immune sites such as the CNS. Indeed, the dysregulation of this bi-directional communication as a consequence of dysbiosis has been implicated as playing a possible role in the pathogenesis of neurological diseases. In multiple sclerosis (MS), the gut-brain axis is increasingly being considered as playing a crucial role in its pathogenesis, with a major focus on specific gut microbiota alterations associated with the disease. In both MS and its purported murine model, experimental autoimmune encephalomyelitis (EAE), gastrointestinal symptoms and/or an altered gut microbiota have been reported together with increased intestinal permeability. In both EAE and MS, specific components of the microbiota have been shown to modulate both effector and regulatory T-cell responses and therefore disease progression, and EAE experiments with germ-free and specific pathogen-free mice transferred with microbiota associated or not with disease have clearly demonstrated the possible role of the microbiota in disease pathogenesis and/or progression. Here, we review the evidence that can point to two possible consequences of the gut-brain axis dysfunction in MS and EAE: 1. A pro-inflammatory intestinal environment and “leaky” gut induced by dysbiosis could lead to an altered communication with the CNS through the cholinergic afferent fibers, thereby contributing to CNS inflammation and disease pathogenesis; and 2. Neuroinflammation affecting efferent cholinergic transmission could result in intestinal inflammation as disease progresses.

## Introduction

Multiple sclerosis (MS) is an inflammatory demyelinating disease of the central nervous system (CNS), the etiology of which is still unclear, albeit believed to result from an autoimmune attack on CNS components. MS is highly heterogeneous, with clinical patterns characteristic of each individual patient and no single pathognomonic marker known. Infection has long been investigated as a possible trigger of MS, albeit without concrete evidence for a particular agent. More recently, however, the concept of a pro-inflammatory gut microbiota as a trigger of autoimmunity has arisen, with the possible implication of demonstrated dysbiosis in MS, based on pre-clinical studies in its purported animal model, experimental autoimmune encephalomyelitis (EAE) (1). Here, we review how the possible dysfunction of the gut-brain axis might impact neurological diseases, with particular emphasis on MS.

## What Is the Gut-Brain Axis?

The gut-brain axis acts as a link between the external environment and the CNS. Its main components are the microbiota for its role in gastrointestinal homeostasis, the intestinal barrier that regulates the entry of food and microbial metabolites into the organism, and the sympathetic and parasympathetic arms of the autonomic nervous system, namely the enteric nervous system (ENS) and the vagus nerve, which transmit signals to the brain.

The human gut microbiota comprises 500-1000 bacterial species and an undetermined number of viruses, fungi, and others (2); it is highly heterogeneous between individuals, varying according to age, diet, and other environmental factors, so an “optimal microbiota composition” does not exist (3). However, microbiota composition is quite stable at phylum level, and an alteration in the ratio between different phyla, with the loss of microbiota diversity, has been associated with diseases (2, 4, 5). In homeostatic conditions, the microbiota not only exerts a large number of functions that are related to food digestion and vitamin synthesis, but it also has an important role in the maintenance of intestinal barrier integrity and in the regulation of the immune system (6). The microbiota communicates with the host mainly through bacterial metabolites of dietary substrates such as tryptophan metabolites and short chain fatty acids (SCFA), modification of host molecules such as bile acids, or directly through bacterial components such as lipopolysaccharide (LPS) (7).

The intestinal barrier is a semipermeable mucosa which consists of the mucus, protecting the underlying layers; the intestinal epithelium, mostly composed of enterocytes and minor secretory cell subsets; and the lamina propria, a thin layer of connective tissue that is densely populated by cells of the innate and the adaptive immune system. The intestinal barrier allows the entry of essential nutrients and beneficial molecules from the gastrointestinal tract, while preventing the entrance of pathogens and harmful antigens (8).

**Figure 1:**
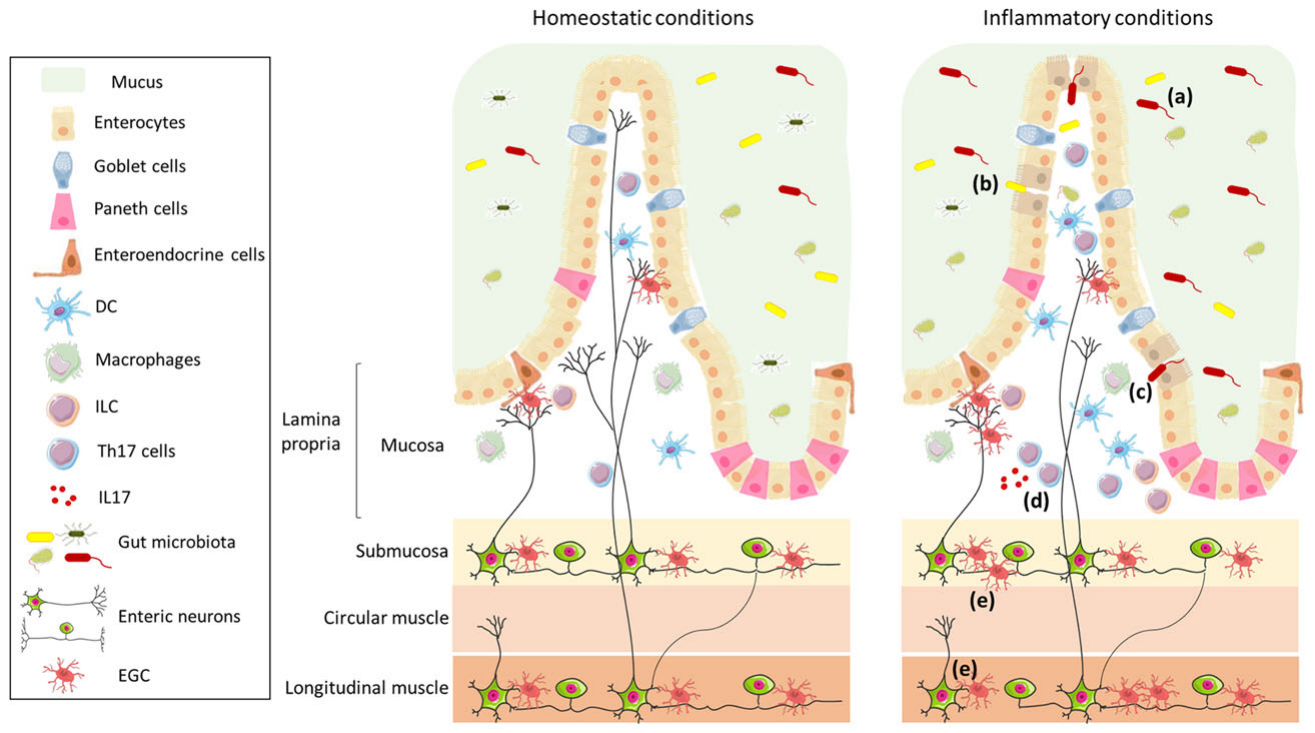
Schematic representation of the intestinal wall in homeostatic and inflammatory conditions. The intestinal barrier is composed of mucus, which acts as physical and biochemical barrier, of a continuous monolayer of intestinal epithelial cells (including enterocytes, goblet cells, Paneth cells, and enteroendocrine cells), and of the lamina propria, which is densely populated by immune cells (such as DC, macrophages, ILC, and T cells). The intestinal barrier is directly exposed to the gut microbiota that has an important role in the maintenance of barrier integrity and in the regulation of the immune system. The lamina propria is innervated by enteric nerve fibres, which originate in the myenteric and submucosal plexi and are in contact with EGC. The left panel shows the intestinal wall under homeostatic conditions; in the right panel, under inflammatory conditions, the composition of the gut microbiota is altered **(a)**, the intestinal epithelial barrier is impaired **(b)**, enabling translocation of microbes (or their products) **(c)**, and resulting in an increase in Th17 cells **(d)** and enteric axonal loss and gliosis **(e)**.

The ENS is the largest part of the peripheral nervous system that innervates the gastrointestinal tract, with nerve fiber endings of the myenteric plexus terminating in close proximity to intestinal epithelial cells (IEC) (**Figure 1**); it is connected to the CNS through the parasympathetic (*via* the vagus nerve) and sympathetic (*via* the prevertebral ganglia) nervous systems. The ENS is composed of an extensive network of neurons and enteric glial cells (EGC), which play an important role in the maintenance of intestinal barrier function (8, 9). ENS functions include the regulation not only of gastrointestinal motility, but also of secretion, nutrient absorption, immune regulation, and defence (10). In particular, glial cell-derived neurotrophic factor secreted by EGC is involved in the regulation of ILC3, which are a component of the glial–ILC3–epithelial cell unit that surveys gut environment and control its defence (11). In a recent study, Yan and co-authors demonstrated that enteric neurons themselves can exert a key role in the regulation of immune cells, by preventing microbial-induced differentiation of regulatory T (Treg) cells through the release of IL-6 (12).

## How the Gut Interacts With the Brain

The bidirectional communication between the gut and the brain occurs in different ways.

### Through the ENS

The ENS is directly connected to the brain through the sympathetic and parasympathetic innervation of the gut. The sympathetic pathway is composed of approximately 50% afferent and 50% efferent nerve fibres, while the parasympathetic innervation of the gut is mainly provided by the vagus nerve, composed of 80% afferent and 20% efferent fibers (13, 14). Many studies have focused on the role of the vagus nerve, as part of the cholinergic anti-inflammatory pathway and for its capacity to sense the gut stimuli and transmit them to the brain (14). Efferent fiber signals from the brain to the gut, in turn, can affect gut motility, secretion, and epithelial permeability, and, through the ensuing change in physical environment, alter the composition of the intestinal microbiota. Of note, vagal efferent fibers also regulate immune responses and cytokine production through the activation of the acetylcholine/α7nicotinic-acetylcholine receptor signaling (15). These fibers innervate the intestinal wall indirectly through synapses contacting the ENS in the myenteric plexus (14), suggesting that central stimulation of the vagus nerve leads to the activation of enteric neurons that subsequently release factors, such as acetylcholine, that might affect the local immune system (16). Vagal afferent fibers can sense a wide spectrum of pro-inflammatory and neuroprotective molecules produced by the microbiota or by the host (cytokines, SCFA, LPS, neurotransmitters, etc…) and thereby transmit gut signals to the CNS. Increasing evidence has shown that oral administration of probiotics, defined as “live microbial strains that beneficially affect the host when ingested in sufficient doses” (17), have a high impact on vagus nerve activity (18, 19). The precise mechanism underlying probiotics-vagus nerve interaction is not fully elucidated. Nevertheless, an increase in the spontaneous firing frequency of the vagal afferent fibers following application of the anxiolytic probiotic *Lactobacillus rhamnosus* has been demonstrated, which was abolished upon vagotomy (20). Thus, oral administration of *L. rhamnosus* reduces anxiety and depression in mice by reverting the expression of γ-aminobutyric acid receptors at CNS level in a vagus nerve-dependent manner (21). Similarly, the administration of another *Lactobacillus* strain (*L. reuteri*) improves social behavior in mouse models of autism spectrum disorder (ASD), which are associated with alterations of the gut microbiota, possibly through stimulation of the vagus nerve. Indeed, treatment of ASD mice with *L. reuteri* led to an increase in the number and fluorescence intensity of oxytocin-positive neurons in the periventricular nucleus of the hypothalamus where vagal fibers project, with release of oxytocin (19) that is known to reverse social deficits in ASD mice (22). Contrasting data were however obtained in another study where administration of *L. reuteri* induces a depression-like behavior *via* the vagus nerve in mice treated with an antibiotic cocktail, suggesting that the effect of *L. reuteri* likely depends on complex synergistic interaction between intestinal microorganisms (23). Altogether, these studies suggest an anti-inflammatory role for vagus nerve stimulation, which was shown to be responsible for modulating microglia activation in aged APP/PS1 mice, a genetic model of Alzheimer’s disease (AD) (24) and to have a positive effect on cognition in AD patients (25). In this context, it has been demonstrated that vagal afferent fibers in the gastrointestinal tract are involved in hippocampal-mediated memory function (26).

### Through Cross-Talk With the Immune System

The immune system represents an important gatekeeper in the gut-brain axis, as it closely interacts with nerves and epithelial cells, and can sense microbiota metabolites (27). The crosstalk between the immune and nervous systems occurs because immune cells express a wide variety of receptors for neurotransmitters/neuropeptides and, in turn, neurons are responsive to cytokines. Besides the anti-inflammatory role of the cholinergic (parasympathetic) system, the sympathetic nervous system also affects intestinal immune cells differently. Indeed, its effects depends on several factors, including the type and the concentration of the neurotransmitter (norepinephrine, adenosine), the affinity of the receptor subtypes on immune cells, and the different functionality of the sympathetic nervous system during different phases of inflammation (28). In this context, macrophages present in the lamina propria or the muscularis express opposite phenotypes, pro-inflammatory *vs* tissue-protective, respectively, at steady state, a divergence that is in part related to norepinephrine signaling *via* β_2_ adrenergic receptors highly expressed on muscularis macrophages (29).

### Through Sensing Microbial Metabolites

Microbiota metabolites can directly and indirectly influence CNS cells. In particular, they regulate microglia development (30, 31) and functions (32). In this context, microglia from germ-free (GF) mice have a dysregulated expression profile of maturation and activation markers, an altered/immature morphology, and a defective response to pathogens. Microbial metabolites, in particular SCFA that include acetate, propionate and butyrate, are involved in the regulation of blood-brain barrier (BBB) permeability. In this context, GF mice have an increased BBB permeability with an altered expression of tight junction, and colonization with specific bacterial strain or the administration of SCFA restores BBB integrity (33).

## Neurological Consequences of a “Leaky” Gut

Pathological or environmental factors directly or indirectly impair intestinal barrier integrity. This can occur through changes in microbiota (34) or upon an increased activation of mucosal immune cells, such as occurs in inflammatory bowel disease (IBD) (35, 36). Dysfunction of this barrier allows the translocation of microbes or their products (such as LPS) from the gut lumen into the lamina propria and from there into the blood, thereby promoting inflammation/an aberrant immune response, not only in the gut, but also at systemic level. Such an increase in intestinal permeability, also known as “leaky gut”, together with an alteration in the commensal population composition, has been associated not only with gastrointestinal disorders, but also with extra-intestinal diseases (37, 38).

In the context of the gut-brain axis, a leaky gut has become an important focus of interest for neurological disorders in the past decade, with the demonstration of intestinal microbiota dysbiosis and/or an impaired intestinal barrier in neurological diseases with different aetiologies, such as stroke (39), Huntington’s disease (40, 41), and amyotrophic lateral sclerosis (42, 43). Intestinal barrier disruption has also been linked to cognitive disorders associated or not with neurodegeneration. In particular, a key role for the gut in the pathogenesis of ASD has been hypothesized with gastrointestinal inflammation reported in as many as 84% of the cases (44). In particular, altered expression of intestinal tight junctions (45), together with an altered composition of the gut microbiota and their metabolic products, have been observed in ASD patients and/or in a mouse model with ASD features (45–47). Interestingly, an increased use of antibiotics during pregnancy seems to be correlated with a higher risk for the development of ASD (47). In this context, administration of an emerging probiotic, *Bacteroides fragilis*, ameliorated gut alterations, dysbiosis, and ASD symptoms in the mouse model (48). While gut dysbiosis is now well established as a possible contributor of AD, both in patients and in several animal models (49–51), there is, to our knowledge, no direct evidence for a dysfunctional intestinal barrier in patients, although it should be noted that intestinal permeability increases with age in the human (52). Interestingly, age-dependent alterations in the gut microbiota associated with gut leakiness are found in the 5xFAD mouse model of AD, with a significant decrease in microbial diversity; transplantation of microbiota from aged AD, but not wild-type, mice accelerated AD pathology in young mice, effects that were mitigated by probiotic bacteria-enriching treatment (50). Similarly, in the ADLP^APT^ mouse AD model, reducing gut dysbiosis through transplantation of faecal microbiota from WT mice improved AD pathogenesis and cognitive impairment (53). In contrast, in another mouse AD model, App^NL-G-F^ mice, administration of probiotics ameliorated intestinal inflammation and leakiness, but not neurological pathophysiology (54). The influence of the microbiota in AD appears related not only to dysbiosis, but also to the ability of amyloids to traverse the gut wall. Gut microbiota produces a high quantity of amyloids in the biofilm that covers the gastrointestinal tract, among which curli is the most studied; it is produced by *Escherichia coli* in stressful conditions (55, 56) and promotes intestinal inflammation (56–58). Interestingly, bacterial amyloids may act as prion proteins, eliciting cross-seeding, through molecular mimicry with amyloid-β (Aβ), *in vitro* and *in vivo*, suggesting that they could induce the formation of Aβ aggregates in the CNS (59, 60). In this context, injection of brain extract containing Aβ in the stomach and gut wall (in the serosa) of the outbred wild-type ICR mice induces CNS amyloidosis and AD‐like dementia by spreading of Aβ through the ENS and vagus nerve (61).

The idea that neuropathology could be triggered by a leaky gut is best exemplified by recent studies in the neurodegenerative Parkinson’s disease (PD) and its experimental model. It stems from studies of Braak et al. in the early 2000’s who suggested that Lewy body pathology could develop first in extra-CNS sites, showing the presence of α-synuclein aggregates both in the olfactory bulb and in the dorsal motor nucleus of the vagus nerve (in the brainstem) (62), as well as in the myenteric and submucosal plexus of PD patients (63). These data led to the hypothesis that α-synuclein aggregates could reach the brain from the ENS, as shown by EGC activation (64), *via* an uninterrupted series of projection neurons, extending from the dorsal motor nucleus of the vagal nerve to the cerebral cortex (63). More recent studies in experimental models support this hypothesis. Thus, the injection of α-synuclein fibrils in the muscle layer of pylorus and duodenum (that are densely innervated by vagus nerve) of wild-type mice leads to pathologic propagation of α-synuclein to the CNS *via* the vagus nerve, resulting in loss of dopaminergic neurons and PD-like clinical symptoms (65). Hence the hypothesis that α-synuclein may originate in the ENS and go to the brain *via* retrograde axonal transport through the vagus nerve (66, 67). Interestingly, in PD patients, gastrointestinal manifestations start years (decades) before the clinical onset of disease and are associated with increased intestinal permeability and dysbiosis (68). Also corroborating this hypothesis is the observation that intestinal infection with Gram-negative bacteria triggers PD-like symptoms in the Pink-/- mouse model of PD (69).

That a leaky gut could indeed lead to neurologic manifestations is also clearly corroborated by the noted observation that IBD is often associated with CNS demyelination, the hallmark of MS (70, 71); conversely, neurological disorders are often associated with gastrointestinal symptoms (72).

## The Gut-Brain Axis in MS and EAE

### Gut Dysbiosis

The possible implication of gut dysbiosis in MS was first studied in EAE through the use of GF mice or antibiotic treatment, which demonstrated that the microbiota plays a crucial role in directing both pro- and anti-inflammatory immune responses in the CNS (73). The importance of microbiota in the pathogenesis of EAE is supported by studies in which the prophylactic (but not therapeutic) antibiotic treatment protects against the disease, possibly by increasing Treg- and Th2-cell responses (74–76). Importantly, antibiotic treatment impaired the development of EAE if given per os specifically, rather than intravenously (76). EAE experiments with mice treated with specific antibiotics or GF mice monocolonized with particular bacteria have clearly demonstrated that signals from an altered gut microbiota can induce inflammation in extra-intestinal tissues (74, 75, 77, 78). This concept has been further elaborated through experiments in which microbiota from MS patients were tested for their ability to modulate EAE. Several studies have demonstrated a dysbiotic gut microbiota in MS, albeit with variables findings (73). A more recent analysis showed significant association with MS of several *Acinetobacter* species, which are rare in healthy human gut, together with the decreased presence of *Parabacteroides*, more particularly *P. distasonis*. Interestingly, exposure of lymphocytes from healthy individuals to MS microbiota or extract from MS-associated *Acinetobacter calcoaceticus* increased their differentiation to Th1 type cells and reduced the proportion of CD25+FoxP3+ Treg cells, whereas exposure to *P. distasonis* extract could skew their T-cell phenotype to regulatory (79).

### Gut-Brain Cross-Talk

EAE experiments further corroborated the pro-inflammatory environment imparted by MS microbiota. Thus, EAE severity in mice pre-colonized with faecal MS microbiota was significantly greater than in mice colonized with control microbiota, and was associated with decreased IL-10+ Treg-cell induction in mesenteric lymph nodes (79). Similarly, colonization with MS microbiota of transgenic mice bearing the T-cell receptor (TCR) for an encephalitogenic epitope significantly increased the spontaneous incidence of EAE in these mice (80). However, the gut environment can also favor the generation of Treg cells that can improve EAE. Indeed, not only effector T cells (81), but also Treg cells are activated within the gut mucosa in response to commensal dysbiosis. In this context, a population of autoreactive CD4+ intraepithelial lymphocytes shown to proliferate in mouse GALT and gut lamina propria in response to non-self-antigens derived from the intestinal contents, infiltrated the CNS upon adoptive transfer to regulate inflammation, decrease demyelination, and ameliorate disease severity (82). Not only T cells, but also B cells reactive to gut commensals could be involved in the cross-talk between gut and brain. Thus, in MS patients, IgA+ B cells specific for MS-associated immune-stimulatory bacterial strains traffic to the inflamed CNS where they colocalize with active lesions (83). As these CNS-infiltrating IgA+ B cells recognizing gut microbiota did not cross-react with brain antigens and were associated with *IL10* expression, the authors suggest that these gut-originating IgA+ B cells represent regulatory cells actively recruited to the inflamed CNS through inflammation-dependent factors, independently of their reactivity to microbial antigens (83).

Altogether these studies clearly demonstrate the importance of the microbiota composition in determining the inflammatory environment in the gut, and its consequences on the CNS. It is highly relevant to MS that dysbiosis in patients is frequently associated with an increased intestinal permeability (84) and both alterations have been also demonstrated in EAE (85–87). That such conditions might promote a systemic inflammatory condition is supported by the increased plasma levels of LPS observed in both MS patients and EAE- affected mice (88, 89), as well as by the increased anti-microbiota systemic IgG responses observed in MS patients, which suggest an enhanced bacterial translocation from the gut lumen to systemic circulation in MS (90).

### The ENS Link

In view of the close proximity of ENS neuronal endings to gut luminal contents, it is likely that the gut microbiota impacts enteric neurons, as demonstrated by the changes in electrophysiological properties of myenteric neurons (91) and intestinal motility (92) in response to alterations of the microbiota, providing a potential link between the microbiota and the CNS. Although the ENS has been poorly studied in MS, gastrointestinal motility disorder is an additional burden in MS (93), and two recent studies suggest that the ENS is involved. Thus, signs of ENS degeneration, including gliosis and axonal loss in the myenteric plexus, which were antibody-mediated and caused significant decrease in intestinal motility, were observed in a B cell- and antibody-dependent mouse EAE model before the onset of CNS disease; myenteric plexus alterations with nerve fiber loss and enteric glia activation were also detected in colon resectates from two of three MS patients analysed (94). Serum autoantibodies against potential target antigens derived from enteric glia and/or neurons were identified in both EAE-affected mice and MS patients (94). Similar observations were made in three other mouse EAE models where autoantibodies targeting ENS components were implicated in altered gastrointestinal motility (95).

### Restoring Gut Homeostasis: Probiotics and Fecal Microbiota Transplantation

The clear involvement of the gut-brain axis in MS pathogenesis suggests that it could be a potential therapeutic target. Accordingly, in view of the profound impact dysbiosis could have on MS, the use of probiotics has been proposed as a potential therapy. Probiotics are thought to contribute to maintaining or restoring a balanced and diverse microbiota (96). Although their mode of action is not fully understood, increasing evidence indicates that they have a high impact on vagus nerve activity (18). Several studies have shown that both the preventive and the therapeutic administration of *Bacteroides fragilis* can suppress EAE by promoting Treg-cell function (97, 98). This anti-inflammatory effect is apparently mediated by polysaccharide A (PSA), a component of *B. fragilis* capsula, which, as a ligand for TLR2, induces the differentiation of Treg cells (99). Oral treatment with PSA induced an increase in the number of CD103+ dendritic cells (DC), a subset of DC involved in the differentiation of Treg cells, in cervical lymph nodes of treated mice (98). Similarly, the preventive per os administration of *Escherichia coli* strain Nissle 1917, a probiotic used for the treatment of many gastrointestinal disorders (100), ameliorates EAE outcome by modulating cytokine production and migration of autoreactive CD4+ T cells and preventing EAE-induced intestinal alterations (101). The combination of different strains of probiotics could exert synergistic therapeutic effects; thus, three strains of *Lactobacillus*, each singly shown to be effective in preventing the development of EAE when administered before disease onset, were shown to inhibit established disease when administered as a therapeutic mixture (102). In MS patients, the administration of a mixture of probiotics (enriched with *Lactobacillus*, *Streptococcus* and *Bifidobacterium*) induced a switch in the peripheral immune response to anti-inflammatory, and reverted the alteration in microbiota composition associated with MS (103, 104). Whether or not these changes were associated with clinical improvement was not assessed in this short-term study, but the data from randomized double-blind placebo-controlled clinical trials of three- and four-month duration with similar probiotic mixtures (*Lactobacilli* and *Bifidobacteria*) suggest that daily probiotic supplementation could ameliorate clinical symptoms of MS (as judged by a small but significant improvement in the expanded disability status scale) (105). However, recent studies in EAE suggest that, in the design of probiotic treatment, it is important to consider that probiotic strains could exert different, even opposite, effects depending on the timing of exposure, the microbiome composition of the recipient, and the recipient’s genetic background. This is best exemplified by the divergent effects of treatment of EAE with *L. reuteri*. Thus, despite its beneficial effect in suppressing the development of the disease when administered before the onset of the symptoms (106), *L. reuteri* within a probiotic mixture could instead contribute to CNS autoimmunity, through potential mimicry with an encephalitogenic protein leading to activation of encephalitogenic T cells in the small intestine (74). In studies of EAE in consomic mice where transfer of the most divergent microbiota from these mice into genetically identical recipients showed that genetically determined gut microbiota modulates EAE severity, abundance of *L. reuteri* in the transferred microbiota, or through its stable introduction in the microbiota associated with the less severe disease, was seen to co-segregate with exacerbated disease (107).

In another approach to therapy targeting the microbiota, humanized HLA-transgenic mice induced for EAE were gavaged before disease onset with *Prevotella histolica*, a commensal decreased in the microbiota of MS patients as compared to that of healthy subjects, but increased in microbiota of MS patients receiving disease-modifying therapies. The treatment restored the gut microbiota to that preceding the encephalitogenic challenge and resulted in reduced gut permeability and BBB dysfunction, with reduced CNS inflammation; disease amelioration was associated with an immunomodulatory effect at systemic level, with a decrease in Th1- and Th17-cell levels and increased frequencies of Treg cells (108).

Restoring gut homeostasis thus appears as a potentially important aspect of the therapeutic approach to MS. In this context, fecal microbiota transplantation (FMT), which has given promising results in IBD (109), is being considered as a possible approach to reestablish gut microbiota composition. In mice induced for EAE, daily gavage with fecal supernatant from naïve mice delayed disease onset and reduced disease expression at both clinical and neuropathological levels, preventing BBB impairment and reducing neuroinflammation, demyelination and axonal loss; the beneficial effect was associated with a trend towards a restored gut microbiota diversity in the treated mice (110). In MS, however, studies to assess the possible effect of FMT are scarce. Serendipitous findings from three MS patients treated with 5-10 FMT infusions for severe chronic constipation demonstrated not only resolution of the condition, but also progressive neurological improvement (111). A recent proof-of-concept single-subject longitudinal study was conducted to evaluate the potential impact of FMT on relapsing-remitting MS. The patient received FMT infusions from five healthy donors and was followed over 12 months for clinical assessment, fecal microbiome composition, fecal SCFA concentrations, and serum levels of inflammatory and neuroprotective biomarkers (112). The treatment resulted in an improved microbiome with increased bacterial diversity partly due to increased relative abundance of butyrate-producing bacterial species, that was accompanied by an increased concentration of the anti-inflammatory SCFA, butyrate. The modified microbiota was associated with decreased levels of inflammatory cytokines and increased levels of the neuroprotective molecule, brain-derived growth factor, in the serum. At clinical level, the patient showed progressive improvement in gait and walking and balance metrics over the course of the study (112). Similarly, a case report indicated that treatment with FMT for *Clostridum difficile* enterocolitis in a patient with secondary progressive MS was associated with disease stability (113). Albeit limited, these findings argue for further investigations of the potential benefit of rebalancing the microbiota through FMT as an adjunct approach to MS therapy, and clinical trials to this effect are ongoing (114).

### Modulation of the Gut Microbiota Through the Diet

Another strategy to modulate the gut microbiota is through the diet, since it is considered the main factor that shapes its composition. A vegetarian diet promotes an increase in the number of SCFA-producing bacteria, leading to immunomodulatory effects not only in the gut but also at systemic level (See above) (115). In this context, preventive, but not therapeutic, treatment by daily gavage with propionic acid ameliorated EAE at both clinical and pathological levels by inducing the differentiation of Treg cells in the small intestine (116). Interestingly, propionic acid is reduced in serum and faeces of MS patients in association with an altered microbiota, and dietary supplementation for three years with this SCFA resulted in clinical and pathological amelioration in the treated patients. This was associated with a rebalancing of the Treg/Th17 cell ratio towards a more regulatory profile and an upregulation of genes related to induction of Treg cells in the intestine (117). The induction of Treg cells by SCFA can occur directly or indirectly by enhancing the production of retinoic acid by CD103+ DC, which drives the differentiation of FoxP3+Treg cells (118, 119). In particular, it has been shown that the anti-inflammatory effect of the commensal butyrate occurs through activation of its receptor, hydroxycarboxylic acid receptor 2 (HCAR2), which triggers an increased production of retinoic acid and IL-10 in intestinal DC (120), modulates the over-activation of ILC3 (121), and decreases pro-inflammatory gene expression in IEC (122, 123). SCFA can also affect B cells, as recently demonstrated in a mouse model of arthritis, in which gut-derived acetate was shown to promote the differentiation of anti-inflammatory IL-10-producing B-cells (124). Of note in this context, serum levels of butyrate correlated positively with the percentage of IL-10-producing B cells and levels of acetate correlated negatively with TNF production by IgM+ B cells in patients with MS or clinically isolated syndrome preceding MS (125).

Other dietary modes that can impact CNS integrity through the microbiota, and thereby through the Th17/IL-17 pathway in the gut, include intermittent fasting. Thus, intermittent fasting exerted a beneficial effect on EAE, ameliorating the clinical course and pathology of the fasting mice through an effect partially dependent on their microbiota that led to a decrease in IL-17-producing T cells together with an increase of Treg cells in GALT (126). In this context, it should be noted that long-term calorie restriction seemingly has a health-promoting effect in mice by inducing a balanced gut microbiota, with increased proportion of beneficial bacterial strains involved in the maintenance of intestinal homeostasis (such as *Lactobacillus*) (127). Moreover, the reduction in protein intake, and in particular the omission of the amino acid tryptophan, have been shown to prevent EAE induction by affecting encephalitogenic T cell responses (128). This effect is dependent on the presence of gut microbiota, as dietary tryptophan restriction leads to an enrichment of tryptophan-synthesizing bacterial genera (128).

The influence of the diet on the gut-brain axis in MS and EAE is also exemplified by a recent EAE study based on the observation that MS is less prevalent in countries where the diet includes high amounts of isoflavones, a class of phytoestrogens known for their antioxidant and anti-inflammatory health benefits. The authors of the study observed a more severe EAE course in mice fed a diet devoid of isoflavones whereas a diet rich in isoflavone was associated with an ameliorated EAE course as compared to the standard mouse chow. The beneficial effect was associated with decreased activation and proliferation of the encephalitogenic T cells and with reduced immune cell infiltrates in the CNS. As shown by the difference in gut microbiome composition between mice fed an isoflavone-rich diet and those fed an isoflavone-free diet, disease amelioration was dependent on the presence of isoflavone-metabolizing bacteria and their metabolites, which, interestingly, are reduced in MS patients (129).

## What Comes First in MS, Gut or CNS Inflammation?

Although gastrointestinal manifestations are common in MS (130) and possible indication can be obtained from retrospective studies in MS patients, such as when comorbidity with IBD occurs or demyelination consecutive to IBD is observed, it is as yet impossible to ascertain which of gut or brain inflammation comes first in MS. In EAE, intestinal barrier permeability, together with morphological alterations, was observed already at 7 days post-immunization (85), prior to clinical disease onset, and was associated with an increase in potentially pathogenic T cells infiltrating the gut lamina propria. However, it has not been assessed at earlier post-immunization times, nor correlated concomitantly with BBB integrity, which is already compromised at this pre-clinical stage (Kerlero de Rosbo and Cedola, unpublished data).

### The Gut Environment Drives MS Pathogenesis

Highly relevant in this context is the observation that T cells get activated in the gut (74, 131) and intestinal CD4+ T cells can play a role in the initiation of CNS autoimmunity. A recent study linked autoimmunity in a spontaneous mouse model of MS to the expression of the transforming growth factor beta (TGF-β) inhibitor, Smad7, in intestinal CD4+ T cells (132). TGF- β and Smad7 are involved in the regulation of naïve CD4+ T-cell differentiation and Smad7 in particular was shown to drive Th1-cell responses in MS and EAE (133). Overexpression of Smad7 in the T cells of these mice led to highly increased disease parameters at all levels, with disease dependent upon induction of the CD4+ T cells at the intestinal mucosal barrier before their migration to the CNS (132). Adoptive transfer EAE experiments showed that only gut-derived T cells or T cells treated to induce expression of gut-homing receptors were able to trigger disease upon transfer in wild-type recipients. Of note, EAE induction was dependent on the presence of a full gut microbiota (132). At the human level, the analysis of intestinal tissue biopsies from 27 MS patients and 27 control individuals revealed an altered TGF-β-dependent Th-cell differentiation pattern in the lamina propria with increased frequencies of inflammatory T cells and reduced FoxP3 expression (132), a process akin to the defective TGF-β1/Smad signaling in IBD (134).

Interestingly, it has been suggested that the levels of circulating mucosal-associated invariant T cells, which are abundant in gut lamina propria, may reflect disease activity in MS, with reduced numbers observed during exacerbation correlating with increased MRI activity in some patients, while at remission increased numbers coincided with decreased MRI activity (135). As recently demonstrated, T cells specific for an encephalitogenic epitope might be expanded and activated by the small intestine microbiota, albeit without evidence that these cells could migrate to the CNS (74). Another recent study in adoptive transfer EAE, which showed the infiltration of encephalitogenic Th17 cells in the lamina propria at a time when no such cells were detected in the CNS, demonstrated that the encephalitogenic cells could later be traced to the CNS of mice with clinical symptoms while they decreased in the gut; blocking the initial transit of the autoreactive T cells to the gut resulted in reduced disease severity (131). In the context of gut inflammation, an interesting study showed that the lack of disease expression in mice lacking IL-17 was not related to the encephalitogenic potential of the T cells, which were fully active upon transfer in wild-type mice, but rather to the absence of IL-17 in the gut. Restoring the microbiota of IL-17-deficient mice to that of IL-17-sufficient mice resulted in full-blown EAE, indicating that the reduced susceptibility to EAE in IL-17-deficient mice was strongly associated with the altered microbiota observed in these mice, suggesting that IL-17 in the gut can modulate the intestinal microbiota (136). This study demonstrated the crucial impact of intestinal IL-17 on microbiota composition and thereby on EAE expression, as reintroducing IL-17 expression specifically in IEC of IL-17-deficient mice resulted in a restored microbiota (136). Highly relevant in this context are the observations that, in MS, there is an expansion of Th17 cells in the intestine, which is associated with microbiota alterations and correlates with high disease activity (137).

**Figure 2:**
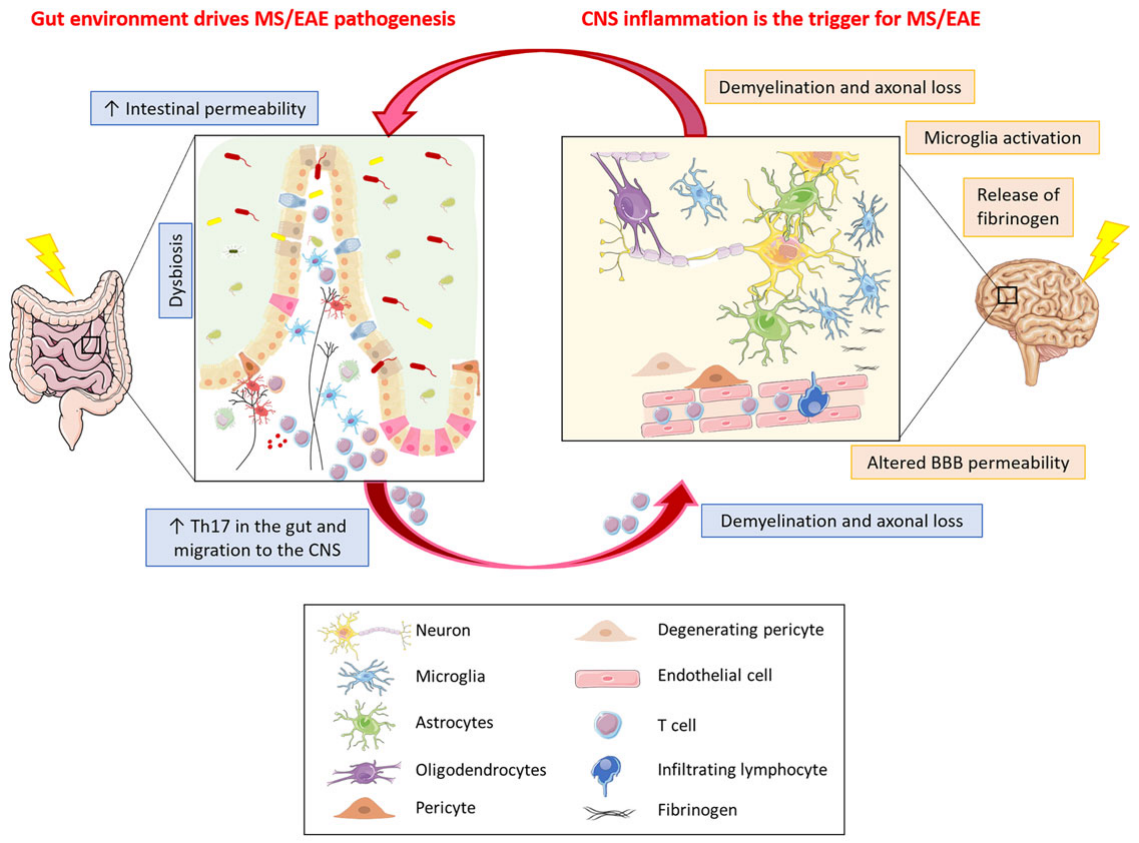
What comes first in MS, gut or CNS inflammation? Gut environment drives MS/EAE pathogenesis. In MS/EAE, dysbiosis together with an increased intestinal permeability, possibly due to alterations in intestinal epithelial barrier morphology, have been demonstrated. These alterations allow the translocation of microbes or their products from the gut lumen into the lamina propria, thereby promoting inflammation. Inflammation in the gut can result in activation of encephalitogenic T cells that can travel to the CNS where they can induce inflammatory damage with subsequent demyelination and axonal loss. CNS inflammation is the trigger for MS/EAE. Neuroinflammation in MS/EAE could be the consequence of an altered vascular permeability of BBB. The leakage of plasma components, such as fibrinogen, leads to rapid activation of microglia that might be the trigger of the disease, with ensuing infiltration of homing inflammatory cells and signal transmission to the gut resulting in intestinal barrier permeability thereby propagating the brain-gut inflammation loop.

It therefore seems that inflammation in the gut can indeed result in activation of encephalitogenic T cells that can travel to the CNS where they can induce inflammatory damage with subsequent demyelination and axonal loss (**Figure 2**). In addition, inflammation in the gut can also influence microbiota diversity and can lead to dysbiosis, which can itself propagate intestinal inflammation. A possible trigger for intestinal inflammation, through dysbiosis, could be the diet, which can alter both the number and the diversity of microbiota species. Studies have shown that a diet rich in salt could be associated with a higher risk for development of EAE and dementia, *via* the induction of Th17 cell differentiation; in particular, mice fed a high-salt diet developed neurovascular and cognitive impairments through the expansion of Th17 cells in the small intestine, leading to an increase in IL-17 in the plasma (138, 139). This increase in Th17 cells is possibly due to a diet-dependent shift in microbiome composition (140) and is induced by the activation of serum glucocorticoid kinase 1 that regulates Na^+^ intake (141). Studies in MS patients demonstrated that a high salt diet induces an increase in Th17 cells in the blood and a decrease in intestinal *Lactobacillus* strain, and is associated with worsened clinical symptoms and enhanced radiological activity (140, 142). However, contrasting data have been reported in MS, where in a five-year clinical trial high levels of urinary sodium in patients with clinically isolated syndrome did not correlate with increased rates of conversion to definite MS or with clinical worsening and/or enhanced MRI activity (143). Similarly, in EAE a high salt diet suppressed the opticospinal encephalomyelitis mouse model by promoting the tightening of the BBB, possibly *via* increasing the serum level of corticosterone (144).

Although EAE experiments in mice transferred with microbiota from EAE-affected mice or MS patients have shown that transfer with a dysbiotic gut microbiota increases disease severity and conversely, there is as yet no concrete evidence that microbiota dysbiosis and its potential inflammatory consequences on the gut-driven immune response are by themselves sufficient to cause EAE or MS. However, in addition to its “non-specific” effect related to inflammation, the microbiota could be involved in another route leading to brain inflammation. Thus, a recent EAE study showed that T cells specific for an encephalitogenic peptide can be activated through mimicry with microorganisms in the small intestine. *Lactobacilli* produce many peptides that have molecular mimicry with the TCR- binding residues of the encephalitogenic peptide, myelin oligodendrocyte glycoprotein (MOG)35-55; in particular, a specific peptide produced by *L. reuteri* induces proliferation of MOG-specific CD4 T cells (74). Although the *L. reuteri* peptide was not by itself encephalitogenic, these findings suggest that molecular mimicry with gut microbiota elements can amplify a pathogenic autoimmune T-cell response.

### CNS Inflammation Is the Trigger for MS

Molecular mimicry with microbial proteins is in fact one of the most common etiological hypotheses for pathogenic autoimmunity, including MS. Indeed, there are many examples of autoimmune disease development following an infection and epidemiological data indicate that viral infections are a risk factor for MS (145). Molecular mimicry is dependent on TCR degeneracy, a common feature of antigen recognition, whereby a single TCR is able to recognise a multiplicity of peptides (146). Such degeneracy has been documented for self-reactive T cells (145, 146) and an early seminal study (147), later confirmed (148, 149), demonstrated that viral peptides can activate human T cells specific for myelin protein epitopes derived from patients with MS. Moreover, cloned MS-derived T cells specific for a disease-relevant human myelin epitope could be activated by microbial peptides subsequently shown to induce EAE in humanized mice transgenic for the relevant human TCR (150). Autoreactive T cells that express a low-avidity TCR bypass clonal deletion (151) and T cells with specificity to myelin epitopes are present in healthy humans (152, 153) and naive wild-type mice (154). Such self-reactive T cells have been shown to respond to their self-antigen or its variants during infection, where inflammation and high levels of antigen presentation prevail (155, 156). It should be noted that active EAE does not develop in the absence of a strong inflammatory Th1 response induced by *Mycobacterium tuberculosis*. These findings suggest that infection could trigger a strong autoimmune response by degenerate myelin-reactive T cells through mimicry with the infectious agent. Interestingly, T cells expressing degenerate TCRs are present at higher frequency in patients with MS (157). Such activated myelin-reactive T cells could then home to the brain to induce inflammation and tissue damage. Indeed, T cells primed in the periphery can bypass the BBB (158), and it is clear that in both MS and EAE, peripherally activated T cells home to the CNS, provided that they recognise an antigen expressed, either inherently or through transgenic manipulation, in that organ (159, 160). In addition, in the last few years, the demonstration of meningeal lymphatic vessels (161) has provided a possible link between the CNS and the peripheral immune system that could be highly relevant to the development of an inflammatory CNS disease such as MS. Thus, CNS antigens collected through the glymphatic system from brain parenchyma into the CSF, could be transported by meningeal lymphatics (162) into cervical lymph nodes with the potential to induce an autoimmune response (161). In this context, DC and T cells, which are found in healthy meninges, can migrate to cervical lymph nodes *via* meningeal lymphatics under both normal and pathological conditions (161). It is therefore possible that, under inflammatory conditions, potentially autoreactive degenerate T cells could initiate an immune response to a relevant CNS antigen presented by DC in cervical lymph nodes. Interestingly, disruption of the meningeal lymphatics or removal of the cervical lymph nodes resulted in delayed EAE onset (161).

CNS inflammation can also be triggered by leakage from CNS vessels. More particularly, dysfunction of the BBB is a hallmark of MS. Although not performed concomitantly with assessment of the gut barrier, a two-photon microscopy monitoring study of blood vessel integrity in EAE clearly demonstrated that focal transient vessel leaks occur in the cortical gray matter within the first three days post-encephalitogenic challenge (163). Such an altered vascular permeability at this early stage of EAE induction appeared to be an initiating event, rather than a consequence of effector T-cell aggregation together with tissue damage at perivascular lesions, as it was not accompanied with an infiltration of inflammatory cells. However, leakage of plasma components such as fibrinogen leads to rapid inflammatory activation of microglia (**Figure 2**) and has been observed in MS and in EAE induced in marmosets where microglial activation was detected in conjunction with early fibrinogen deposits prior to tissue damage or gadolinium enhancement on magnetic resonance imaging (164). Interestingly, depletion of fibrinogen ameliorates EAE (165). In both EAE and MS, there is evidence that microglia activation might be a trigger of disease. Thus, in EAE it was shown to precede infiltration of inflammatory cells (166) and impairment of microglia reduces EAE severity (167); in MS, the presence of frequent, macroscopically invisible, pre-active lesions, not associated with BBB disruption, suggests CNS-intrinsic factors leading to innate immune activation (168). However, there is no indication that focal vessel leakage such as seen shortly after EAE induction, occurs in the absence of specific barrier breaching events and the nature of possible CNS-intrinsic stimuli of innate immune activation is unclear. In this context, however, dysfunction or degeneration of pericytes, resulting in BBB permeability and accumulation of blood-derived fibrinogen in brain parenchyma could play a crucial role in mediating neuroinflammation (169). Interestingly, in early MS, vascular permeability in white matter tracts is associated with pericyte dysfunction (170), and soluble fibrinogen can trigger pericyte death by autophagy (171). In addition, in view of their close association with the brain vessels, activated pericytes, which express receptors for inflammatory molecules, could propagate inflammation generated elsewhere in the body and release pro-inflammatory factors deleterious to BBB integrity and function (169).

On the other hand, signals from the brain, in particular through the sympathetic and parasympathetic nervous systems and the ENS, can trigger intestinal inflammation and increase intestinal barrier permeability following CNS injury (172). This in turn will lead to gastrointestinal dysfunction, immune cell activation in the gut, and gut dysbiosis, which in turn can result in an increased CNS inflammation.

These different hypotheses, however, are not mutually exclusive.

## Discussion

At this stage, the primary trigger for the dysfunctional gut-brain axis in MS still hides within a chicken-egg causality dilemma, with an apparent self-feeding loop of brain-gut inflammation. Thus, innate immune activation in the brain might itself be triggered by signals from the gut. Metabolites of the gut microbiota affect CNS cells (30–32, 173) and the innate immune response in the CNS was shown to be affected by the microbiota. Indeed, an increased production of SCFA, in particular butyrate, caused by an alteration of the microbiota upon feeding mice a high-fiber diet, resulted in a decrease in inflammatory microglia gene expression (174). Similarly, microbial metabolites of dietary tryptophan controlled by the commensal microbiota act directly on microglia and regulate microglia control of astrocyte-mediated neuroinflammation, *via* the aryl hydrocarbon receptor (173), while gut microbiota-modulated production of IFNγ by meningeal NK cells drives the differentiation of a new subset of astrocytes (characterized by the expression of LAMP1^+^TRAIL^+^) that have an anti-inflammatory function in EAE (175). To shed light on the implication of gut dysbiosis in diseases like MS, it will be necessary to clearly define the sequence of events that lead to intestinal barrier dysfunction *vs* brain barrier dysfunction and delineate the timing at which these first occur. This could be done in EAE, but is nearly impossible in MS where disease initiation is unclear and could occur many years before actual symptoms drive the diagnosis. Obviously, EAE is not MS and the mechanisms that underlie the deleterious T-cell response might totally differ, with gut events promoting MS, while CNS events promote EAE, or vice versa. Regardless, it is clear that the gut-brain interaction is of utmost importance in the progression of the disease and therapeutic approaches which target gut dysbiosis and intestinal barrier dysfunction must be seriously considered as drug adjuncts in the future pharmacopeia of MS.

## Author Contributions

All authors listed have made a substantial, direct, and intellectual contribution to the work and approved it for publication.

## Funding

We gratefully acknowledge the funding by FISR-Tecnopolo per la Medicina di Precisione. D.G.R. n. 2117 of 21.11.2018.

## Conflict of Interest

The authors declare that the research was conducted in the absence of any commercial or financial relationships that could be construed as a potential conflict of interest.

## Publisher’s Note

All claims expressed in this article are solely those of the authors and do not necessarily represent those of their affiliated organizations, or those of the publisher, the editors and the reviewers. Any product that may be evaluated in this article, or claim that may be made by its manufacturer, is not guaranteed or endorsed by the publisher.
